# Pulmonary Vein Thrombosis: A Recent Systematic Review

**DOI:** 10.7759/cureus.993

**Published:** 2017-01-23

**Authors:** Gerard Chaaya, Priya Vishnubhotla

**Affiliations:** 1 Internal Medicine, University of Central Florida College of Medicine; 2 Medicine, Hematology-Oncology, Orlando VA Medical Center

**Keywords:** pulmonary vein, thrombosis

## Abstract

The pulmonary veins (PVs) are the most proximal source of arterial thromboembolism. Pulmonary vein thrombosis (PVT) is a rare but potentially lethal disease; its incidence is unclear, as most of the literature includes case reports. It most commonly occurs as a complica­tion of malignancy, post lung surgery, or atrial fibrillation and can be idiopathic in some cases. Most patients with PVT are commonly asymptomatic or have nonspecific symptoms such as cough, hemoptysis, and dyspnea from pulmonary edema or infarction. The thrombi are typically detected using a variety of imaging modalities including transesophageal echocardiogram (TEE), computed tomography (CT) scanning, magnetic resonance imaging (MRI), or pulmonary angiog­raphy. Treatment should be determined by the obstructing pathological finding and can include antibiotic therapy, anticoagulation, thrombectomy, and/or pulmonary resection. The delay in diagnosing this medical entity can lead to complications including pulmonary infarction, pulmonary edema, right ventricular failure, allograft failure, and peripheral embolism resulting in limb ischemia, stroke, and renal infarction (RI).

## Introduction and background

PVT is rare and underdiagnosed in clinical practice, but it is a potentially serious and life-threatening condition. In an autopsy series, Onuigbo concluded that this entity was underdetected in previous reports [1]. The incidence is unclear, as most of the literature includes case reports. Its rare occurrence is due to a rich network of venous collateral vessels that drain the lung; however, certain clinical conditions can lead to obstruction [2]. PVT has been reported in the medical literature occurring in the early postoperative period following lobectomy and lung transplantation [3-4], and in association with metastatic car­cinoma. Some cases have been described as idiopathic. Symptoms can man­ifest as dyspnea, cough, or hemoptysis. Diagnosis is often difficult and can be missed if there is not a high level of sus­picion. Without proper identification and prompt treatment, peripheral embolization including acute stroke can occur and have catastrophic results. Since PVT is a rare condition with potentially life-threatening complications, we decided to make a comprehensive review of the literature in order to provide an overview that can act as a stepping-stone.

## Review

### Pathophysiology

Potential mechanisms of thrombosis are of a mechanical nature, vascular torsion, or direct injury, which remains the most probable precipitating factor [5]. PVT is more frequent after lung transplantation, involving the pulmonary venous anastomotic site.

**Figure 1:**
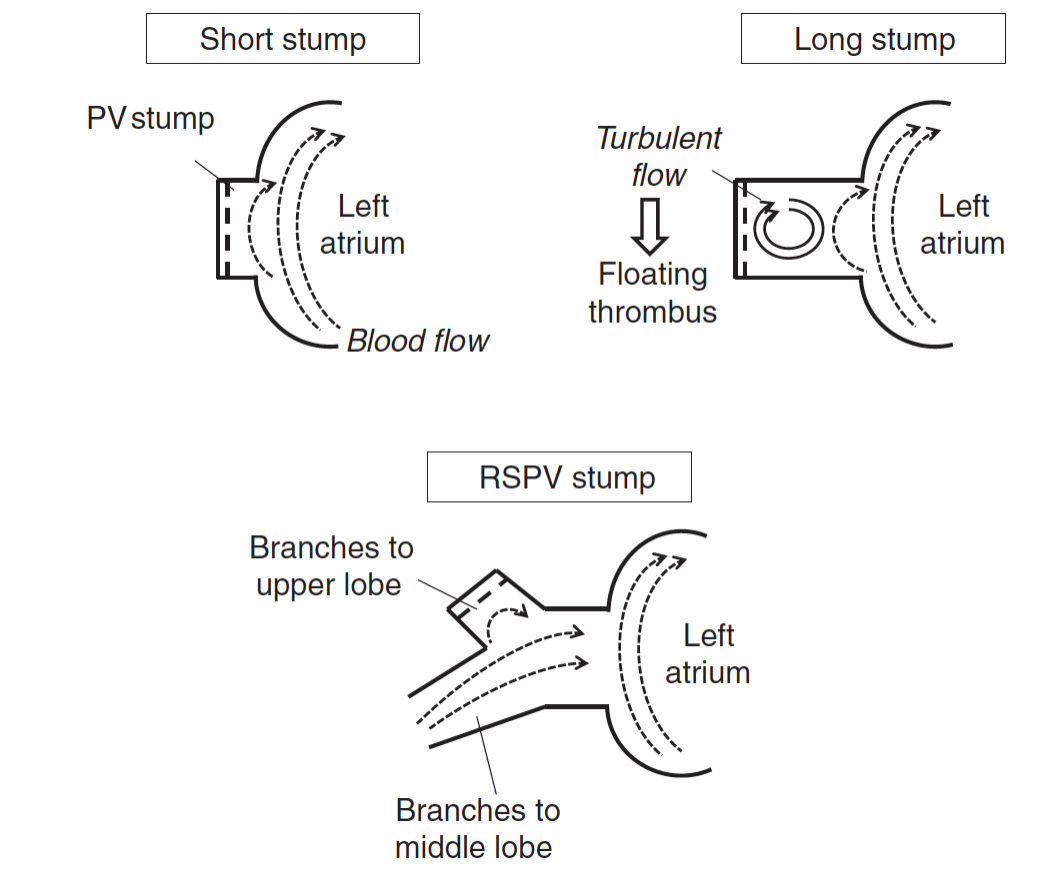
The hypothesis of a thrombus in the pulmonary vein stump after lobectomy In a short PV stump, blood flow may occur because blood flow in the left atrium (LA) spreads through the entire PV stump. In the long PV stump, turbulent flow or stasis of blood may occur because blood flow in the LA does not spread throughout the stump [6].

Ohtaka et al. reported 18 patients with PVT following left upper lobectomy (LUL) [6]. They speculated that the cause of thrombosis in the left superior PV (LSPV) stump after LUL was the long LSPV stump. It might develop because turbulent flow or stasis of blood occurs in the long PV stump [6]. Furthermore, Kwek et al. reported that thrombosis developed in longer PV stumps [6]. In a short PV stump, blood flow may occur because blood flow in the left atrium spreads through the entire PV stump. In the long PV stump, turbulent flow or stasis of blood may occur because blood flow in the left atrium does not spread throughout the PV stump (Figure 1). In the right superior PV, because the branches to the upper lobe and middle lobe remain after right upper and right middle lobectomies, blood flow in the remaining branches spreads throughout the stump, and turbulent flow or stasis of blood may not occur (Figure 1).

The pathogenesis of PVT from a tumor is unclear, although several theories have been postulated. It could result from direct extension of the tumor into the vein, from com­pression of the vein by the tumor, from epithelial damage as a re­sult of tumor invasion, or from a hypercoagulable state [1,7].

### Etiologies

Case reports have identified several causes of PVT, which include surgery involving veins such as lung transplantation or lobectomy; radiofrequency catheter ablation (RFCA) for atrial fibrillation; sclerosing mediastinitis; and certain primary or secondary tumors of the lung. Less common causes include: atrial myxoma; congenital pulmonary venous narrowing; and mitral stenosis with an obstructing left atrial clot [2].

Some authors have categorized PVT causes into primarily postoperative and non-postoperative etiologies [4]. The first report of non-surgical PVT was published in 1925 following the autopsy of a young male with testicular cancer, who died after an acute collapse in the hospital [8].

Lung Transplantation

PVT has been documented in 15% of patients in the early postoperative period (first 48 hours) after lung transplantation [7,9-11]. However, PVT can occur as late as four weeks to two years post transplantation [4]. The reported incidence has declined each year; a finding suspected to be due to improved operative technique [4]. In these cases, patients may present with symptoms and findings caused by obstruction of pulmonary venous flow in addition to thromboembolic events. The mechanism of thrombus formation is likely related to endothelial injury at the time of surgery and blood stasis in the resulting blind PV stump [9]. No donor or recipient risk factors have been identified, such as history of deep vein thrombosis or prothrombotic disorders [4,12]. Some patients received perioperative bovine pancreatic trypsin inhibitor (aprotinin); however, the thrombotic risk with this agent has not been established [12]. In an analysis of 153 lung transplant recipients, 45 patients developed upper or lower extremity deep vein thrombosis. There was a strong association with the presence of an indwelling catheter, infections, and the use of prednisone with mycophenolate mofetil plus either cyclosporine or tacrolimus. In contrast, sirolimus or azathioprine-containing regimens had a markedly lower incidence of deep vein thrombosis [12].

*Lobectomy* 

There have been only six reported cases of PVT after lobectomy [13-17]. All of these cases involved resection of the left upper lobe with subsequent thrombus formation in the left upper PV stump. The true incidence of PVT after lobectomy is unknown and likely underdiagnosed, especially in asymptomatic patients who do not undergo postoperative imaging with TEE or CT [9]. PVT after lobectomy is not a benign finding because thromboembolic complications, including transient ischemic attack and stroke, have been reported. Ohtaka et al. conducted a retrospective study, and they found that thrombosis in the PV stump developed in 3.3% of the patients who underwent lobectomy, and in 17.9% of those who underwent LUL [6]. Clinically, thrombi in the systemic circulation typically develop as a result of atrial fibrillation. It is reported that left lobectomy might be a risk factor for atrial fibrillation. However, the 18 patients reported in the Ohtaka study had no atrial fibrillation when the thrombosis was diagnosed.

Malignancy

The most frequent malignant cause of PVT is a primary lung neoplasm [11], especially bronchogenic carcinoma [1]. However, PVT can also occur following a metastatic cancer [18] such as metastatic sarcoma [11], liposar­coma [18], small cell lung cancer [19], and mantle cell lymphoma of the small intes­tine [7].

Atrial Fibrillation

It is unclear whether atrial fibrillation leading to blood stasis is a risk factor for thrombus development [6]. There is also uncertainty regarding the thromboembolic risk associated with left upper pulmonary vein thrombus in atrial fibrillation, especially in the post-cardioversion period. In the context that atrial fibrillation is increasingly common, ablation catheter procedures have evolved as a new treatment option for drug-refractory atrial fibrillation, and complications of radiofrequency ablation can have important pulmonary manifestations. Series to date suggest that the prevalence of pulmonary vein stenosis after radiofrequency ablation ranges from three to 42%, varying by the method of assessing venous stenosis and the ablation technique used [20]. In the largest available consecutive series, Saad et al. described severe pulmonary vein stenosis (defined as 70% luminal narrowing) in five percent of 335 patients [20]. Total occlusion of at least one pulmonary vein occurred in 2.1% of patients. In another series, Ernst et al. described total pulmonary vein occlusion in 1.3% of 229 patients [20]. Finally, Robbins et al. reported severe pulmonary hypertension in two patients who experienced severe narrowing of all four pulmonary veins near the left atrial junction three months after successful radiofrequency ablation [20].

Other Etiologies

PVT has been occasionally reported after blunt chest trauma [5,21]. It has also been reported in a patient with sickle cell disease [5]; it was hypothesized that the underlying cause was vascular stasis secondary to hypoxia and sickling [9]. One reported case identified large hiatal hernia as a potential cause of PVT by compression of the intrathoracic structures. In a study exploring the physiologic changes associated with a hiatal hernia, direct compression of the left and right inferior pulmonary vein was frequently visualized on contrast-enhanced cardiac CT scans in 12 of 30 (40%) and 11 of 30 (37%) patients, respectively [5].

Idiopathic

Idiopathic PVT has been described in cases of hemoglobinopathy [11,22]. To our knowledge, only two previous cases of spontaneous idiopathic PVT have been reported [23-24].

### Signs and symptoms

Most patients with PVT are commonly asymptomatic or have nonspecific symptoms such as cough, hemoptysis, and dyspnea from pulmonary edema or infarction [1-5,7,11,25]. Hemodynamic signs of PVT are nonspecific and can mimic acute graft rejection (hypoxemia and interstitial infiltrate in the transplanted lung), right ventricular failure, or reperfusion injury [10]. In the majority of cases, pulmonary artery pressure increases concomitant with systemic hypotension and low cardiac output. Respiratory parameters also deteriorate, including worsening oxygenation, hypercapnia, and decreasing pulmonary compliance [26]. In one case, persistent fever was the only symptom [27]. Schiller and Madge noted that three of 16 patients had a systolic murmur in various locations, although the significance of this observation is unknown [1]. Neurologic deficit due to cerebral emboli may be the sole manifestation [5,28].

### Diagnosis

PVT is difficult to diagnose clinically, as reported associated signs and symptoms are nonspecific. In previous cases, the diagnosis was made by either pulmonary angiogram or pathologic examination [3]. Unsuspected pulmonary vein thrombosis discovered during surgery carries a grave prognosis because of the high incidence of massive embolization. Thus, preoperative diagnosis is of vital importance, since alternative techniques of pulmonary venous clamping and cardiopulmonary bypass may minimize the risk of embolization [1]. PVT diagnosis does not pose any particular difficulties in the postoperative period after lobectomy or after lung transplantation; it usually presents a few days after surgery, with sudden-onset dyspnea and chest X-ray studies showing unilateral airspace disease without loss of volume [5]. Furthermore, physical examination and plain chest radiography do not aid in diagnosis [4]. Actually, PVT diagnosis requires a combination of conventional imaging modalities such as pulmonary angiography, transthoracic echocardiography (TTE), TEE that can distinguish between tumor and thrombus, CT after injection of intravenous contrast material in the late phase to reduce flow artifacts, and more recently, MRI [1-2,9,11].

Chest X-ray

Chest radiographic abnormalities include consolidation of the lung and pleural effusion [25]; therefore, chest X-ray studies do not aid in diagnosis.

CT

**Figure 2:**
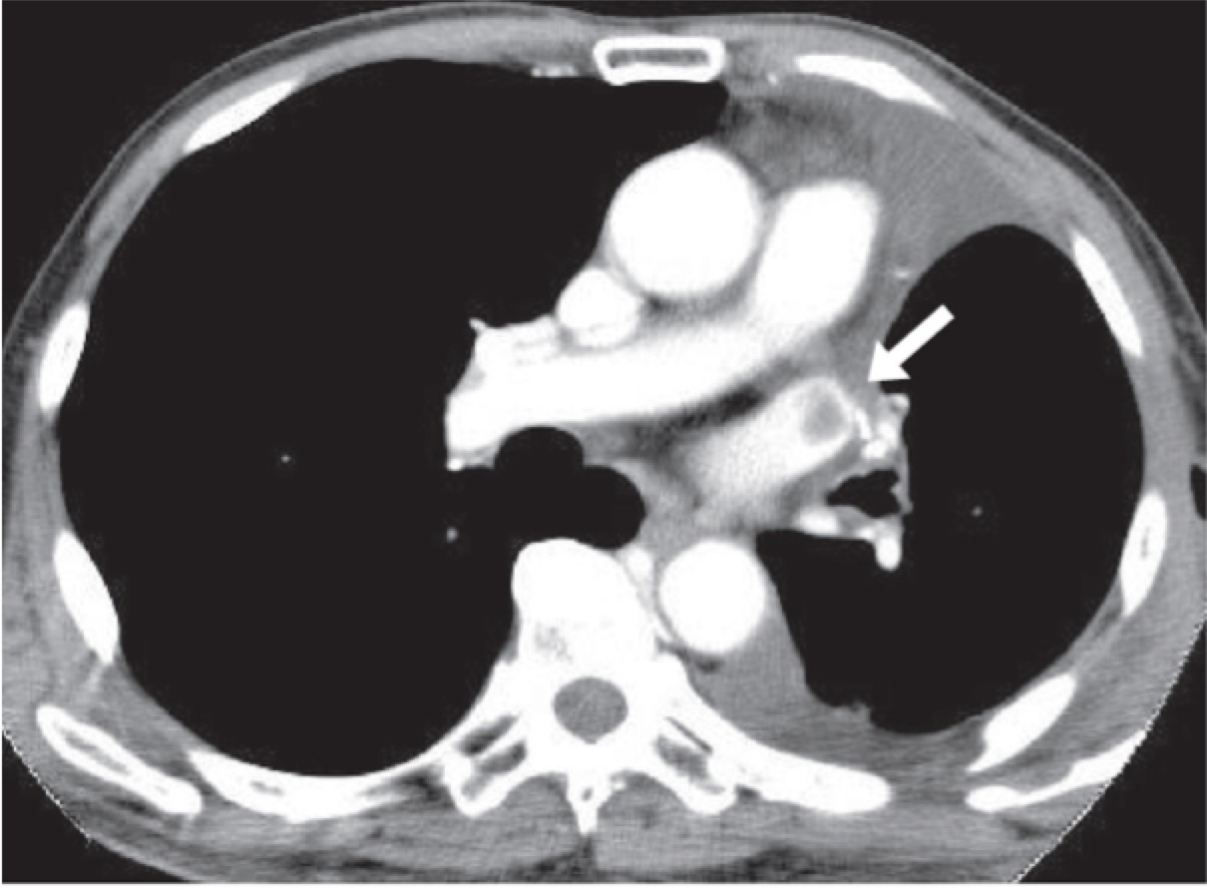
CT scan of the chest with IV contrast A thrombus (white arrow) in the left superior pulmonary vein stump after left upper lobectomy [6].

CT requires intravenous (IV) injection of contrast medium and ionizing radiation. Artifacts from heart motion or concentrated dense contrast medium can cause filling defects to be overlooked within the left atrium [22]. Enhanced helical CT examinations that are tailored to show arterial anatomy may be misleading. Poorly opacified venous blood may cause a true filling defect to be overlooked. Mixing artifacts from opacified and unopacified blood in the atrium may falsely mimic a left atrial mass. A longer scan delay may reduce these artifacts and allow better evaluation of the pulmonary veins and the cardiac chambers [22]. Newer CT techniques have made identifying PVT possible in a similar manner to which pulmonary arte­rial emboli are detected by using the pulmonary venous phase of a con­trast CT of the chest (Figure 2). In a case report of large thrombi in the left lower pulmonary vein, which were connected to thrombi in the left atrium (LA), it was difficult to distinguish small-sized thrombi from artifacts by only TTE [10]. The LA thrombus was confirmed by 64-slice multidetector CT (64-MDCT) scan. Therefore, if small LA thrombus is suspected on TTE, then 64-MDCT may certify it [10-29]. 64-MDCT is superior to TTE for depicting the thrombi within not only the pulmonary vein but also within the LA and can therefore potentially contribute to identifying the LA thrombus. Furthermore, when the thrombus is in contact with the pulmonary vein wall, it is difficult to identify the thrombus in the pulmonary vein by TEE because of pulmonary air, but it is easily detected by 64-MDCT [30].

 

MRI

MRI of the chest is an­other useful modality for diagnosis because it can distinguish be­tween a bland thrombus and a tumor thrombus in the pulmonary vein [22,31]. A bland clot may have persistently high signal intensity on the first and second echoes of spin-echo sequences [31]. Gradient-echo sequences can distinguish extrinsic compression from an intraluminal mass [32]. A tumor may be present when an abnormal signal is seen to extend through the vessel wall [31] or when the intraluminal mass enhances after IV injection of contrast medium.

TEE

**Figure 3:**
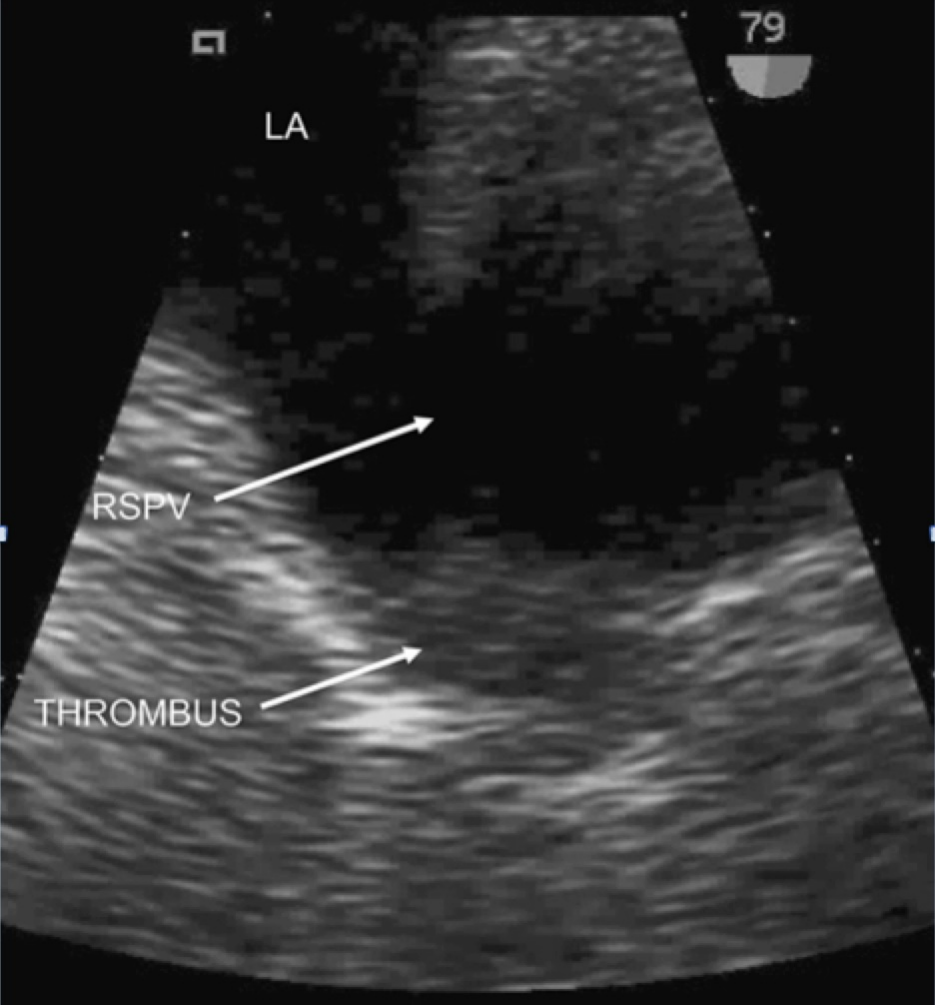
Baseline 2D transesophageal echocardiography TEE demonstrating mobile echogenic thrombus within right superior pulmonary vein. LA = left atrium; RSPV = right superior pulmonary vein; THROMBUS = pulmonary vein thrombus [36].

Echocardiogra­phy may demonstrate the extension of the thrombus into the atrium; a transesophageal echocardiogram would be preferable over a trans­thoracic echocardiogram [7]. TEE can show the thrombus when it extends into the larger distal veins and left atrium [22]. Although not all thrombi can be directly visualized with two-dimensional imaging by TEE, measurement of the blood flow velocities in the pulmonary veins can indirectly suggest this diagnosis (i.e. pulmonary vein blood flow acceleration indicates venous obstruction) [26]. When used intraoperatively, TEE provides real-time two-dimensional imaging of the left atrium and pulmonary veins, allowing for PVT identification (Figure 3). In particular, during sequential double-lung transplantation when systemic anticoagulation and cardiac bypass are not used, the pulmonary vasculature and anastomoses are exposed to longer periods of surgical manipulation, potentially impeding blood flow [26]. TEE can provide early clues of PVT presence and can aid in identifying this potentially catastrophic problem before the patient leaves the operating room. Furthermore, in the intensive care unit setting, postoperatively, TEE can be used to diagnose and follow pulmonary vein thrombosis regression during anticoagulation therapy [26].

Schulman et al. prospectively studied PVT after lung transplantation by assessing pulmonary vein anastomosis using TEE [33]. Eighty-seven consecutive adult lung transplant recipients underwent TEE within 48 hours after surgery. In that study, the overall incidence of PVT was 15%, whereas the usefulness of TEE for detecting significant abnormalities of pulmonary venous anastomoses after lung transplantation was also shown. Schulman et al. found that in lung transplant recipients with PVT, the mean pulmonary venous blood flow velocity was 123 +/-23 cm/s compared with 50 +/-10 cm/s in patients without PVT [33]. A report by Huang et al. also supports the value of intraoperative TEE in early detection of pulmonary venous anastomotic obstruction [34]. Specifically, the authors were able to identify the problem using intraoperative TEE before the patient left the operating room and before clinical symptoms developed, although ultimately the pulmonary vein stenosis was not correctable because of technical issues. Based on the authors’ experience and the limited experience of others as published in the literature, the authors recommend the use of intraoperative TEE as a routine monitor during sequential double-lung transplantation. Leibowitz et al. emphasized the usefulness of TEE for PVT detection after lung transplantation [35].

Given this high frequency of PVT, it could be recommended to perform TEE routinely in the early postoperative phase to exclude the occurrence of PVT, even in the asymptomatic patient. TEE is likely to be more sensitive at identifying right superior pulmonary vein thrombus than pulmonary magnetic resonance venography (MRV) because it allows direct visualization of the lumen and wall of the vessel [36]. The high accuracy of TEE in evaluating posterior cardiac structures such as the pulmonary veins has been established. Some authors suggest that TEE may be the initial diagnostic study of choice for PVT [1].

Furthermore, the best diagnostic test is chosen based on the clinical scenario. For example, TTE may have difficulty carefully observing the PV stump after lung resection, and TEE may be a painful test and more invasive than other tests because it sometimes requires sedation [34]. Though currently uncommonly diagnosed, PVT diagnoses will potentially increase with the rising number of lung transplants, lobectomies, and radiofrequency catheter ablations being performed, but a high index of suspicion remains key to the diagnostic process [2]. TEE may be advantageous in critically ill patients since the procedure is performed at the bedside, and it can quantify the degree of obstruction and direct therapy appropriately. CT scans, however, are less invasive and require less highly trained personnel. MRI, particularly that performed with intravascular gadolinium injection, has been shown to reveal venous thrombosis.

### Management

Treatment of PVT should be determined on the basis of the obstructing pathological finding and can include antibiotic therapy, anticoagulation, thrombectomy, and/or pulmonary resection [2]. Currently, there are no published treatment guidelines or an expert consensus on optimal management. Systemic anticoagulant is frequently initiated, at least until resolution of the clot is observed. In several cases of postoperative patients in whom anticoagulant was contraindicated, spontaneous lysis of small, untreated thrombi without sequelae was observed. Thrombectomy has been successfully performed when medical therapy fails. Lobectomy may be indicated when PVT is complicated with massive hemoptysis or pulmonary necrosis [4].

Antibiotic Therapy

The occurrence of PVT after lobectomy or bilobectomy is potentially life threatening; therefore, antibiotics are generally necessary because of secondary infection of the lung segment involved [25].

Anticoagulation

Management in all cases should include systemic anticoagulation [1,3,9]. Patients who develop PVT following malignancy are usu­ally anticoagulated with therapy for the cancer [7]. Whether anticoagulation should be offered to patients before surgery to reduce thrombus size is unknown. A large dose of heparin administered during the operation as well as postoperative systemic anticoagulation, as Shah et al. suggested, may be necessary to prevent early PVT [10,24]. However, the risk of postoperative bleeding must be considered. Warfarin therapy for PVT has been previously reported [1]. In a case report, dabigatran successfully dissolved a pulmonary vein thrombus [37]. Treatment of PVT depends on the overall clinical condition of the patient. Irrespective of the etiology, a review of the literature does not indicate the preferred duration of anticoagulation or preference for modality of anticoagulation between oral vitamin K antagonists or heparin (low molecular or unfrac­tionated) [18,22,25,33,38]. Both short- and long-term anticoagulation have been utilized successfully in the literature [39]. Clearly, further data are needed to evaluate the risks and benefits of anticoagulation therapy in PVT, but our review of literature suggests that anticoagulation could minimize the risk of embolization in patients with unresectable tumors.

Thrombectomy

Thrombectomy has been tried successfully for thrombosis after lobectomy and lung transplant [10], but few data exist on thrombectomy for PVT due to malignancy [1].

Pulmonary Resection

Failure of clinical improvement, suspicion of gangrene, massive hemoptysis, or pulmonary necrosis should lead to pulmonary resection. Although surgical resection was undertaken due to nonclinical improvement in five of six cases of PVT secondary to lobectomy [25], it may not be necessary in all cases.

Treatment of Postoperative PVT

There is no consensus regarding treatment of postoperative PVT. Therapeutic options range from conservative strategies (anticoagulation and antibiotics) to more aggressive approaches like thrombectomy. However, spontaneous lysis can happen, and spontaneous recovery may be a reason for the rarity of PVT outside the postoperative period [4].

Treatment of Post Lung Transplant PVT

Although the management of lung transplant recipients with PVT is not uniform, the thrombus size and blood flow velocity at the anastomotic site may be used to guide the treatment [33]. A more conservative approach has been advised in the presence of a small non-obstructive thrombus with minimal acceleration of blood flow velocity at the site of the thrombus. In a report by Nahar et al. [40], small pulmonary vein thrombi were successfully managed conservatively on the basis of the thrombus size and the lack of accelerated blood flow at the level of the thrombus as assessed by TEE. Systemic heparinization has been recommended for medium or large thrombi exhibiting elevated peak blood flow velocities. Larger, obstructive thrombi may require surgical thrombectomy [10]. A case of successful thrombolysis by recombinant tissue plasminogen activator has also been reported [41]. Although there have been several reports of patients undergoing emergency thrombectomy [10,24,42-43], unfortunately, most of them succumbed.

Treatment of Post Malignancy PVT

Accurate diagnosis is important to minimize embolization in surgically resectable tumors. In unresectable cases, anticoagulation may be an important adjunct to antitumor therapy [1].

No studies have been conducted regarding management of PVT, but anticoagulation, antibiotics, and in cases of large PVT, thrombectomy or pulmonary resection in resectable tumors have been used.

### Complications

Complications commonly associated with PVT include pulmonary infarction, pulmonary edema, right ventricular failure, and allograft failure [4]. Although less commonly reported, peripheral embolism can occur and has resulted in limb ischemia, stroke [40,44-45], and renal infarction.

Cerebrovascular Accident

Pul­monary vein thrombosis has been reported to result in systemic em­boli, resulting in cerebrovascular ac­cidents [1,7,12-13,15,22]. A review of the literature demonstrates fairly few case reports highlighting stroke and systemic embolization from PVT. There have been no randomized control trials to date. A study by Grau et al. in 2002 evaluated multiple patients with cryptogenic stroke for PVT by MRV and did not find PVT to be a significant contributor to the etiology of ischemic stroke in these patients [45]. However, the study was significantly limited by frequent inadequate visualization of the left pulmonary veins due to limitations in MRI technique. Perhaps with the improved radiologic techniques developed over the last 10-15 years, more cases of PVT could be discovered, particularly in the left side of the pulmonary venous system.

Allograft Failure

Furthermore, acute PVT occurring postoperatively in the lung transplant patient may be disastrous and lead to early postop­erative allograft failure (related to obstruction of pulmonary venous flow causing severe pulmonary edema) [7,26]. In a prospective study, 87 consecutive adult lung transplant recipients underwent TEE within two days after surgery [12]. PVT was diagnosed in 13 (15%) of 87 patients in the early postoperative period. Five (38%) patients with PVT died during the perioperative period. Three of the five deaths resulted from graft failure. In another report, a 90-day mortal­ity of 38% was reported following lung transplant [33].

RI

Renal infarction has been described as a complication of PVT. To the best of our knowledge, only six cases of RI following lung resections have been reported, and none of these cases had detectable well-known causes of RI [46]. Two patients who suffered from RI had a graphically confirmed PVT. The remaining four patients who had RI in the early postoperative period were diagnosed as idiopathic RI, which may be correlated with the postoperative and/or paraneoplastic hypercoagulable state. However, the coexistence of thrombus in the pulmonary vein was not examined in three cases and not mentioned in one case because PVT has not been previously described as a risk factor for RI. In addition, the occurrence of thrombus formation in the stump of the LSPV is more frequent than presumed previously. Therefore, a history of left upper lobectomy should be considered as a potential risk factor for RI, and pulmonary vein thrombosis needs to be regarded as an origin of thromboembolism to make an accurate causal diagnosis of RI.

Other Rare Complications

Other complications that are frequently encountered in idiopathic PVT include pulmonary gan­grene, peripheral embolus, and massive hemoptysis [11,22,47].

## Conclusions

Pulmonary vein thrombosis pres­ents in a nonspecific manner. The diagnosis is now more readily made with the advent of a variety of di­agnostic modalities, especially with transesophageal echocardiography, which may be performed at the bed­side in the intensive care unit. A diagnosis of PVT needs to be con­sidered in patients with appropriate risk factors; for example, in patients with cryptogenic stroke or systemic emboli, it is reasonable for clinicians to evaluate for PVT. An early definitive diagnosis is paramount, which plays a key role in the ability to successfully “rescue” the patient and in the prevention of severe complications. The treatment remains challenging with mortality dependent on the etiology.
